# Isotopic Exchange between Aqueous Fe(II) and Solid Fe(III) in Lake Sediment—A Kinetic Assemblage Approach

**DOI:** 10.1021/acs.est.4c07369

**Published:** 2025-03-11

**Authors:** David W. O’Connell, Catherine Mccammon, James M. Byrne, Marlene Mark Jensen, Bo Thamdrup, Hans Christian Bruun Hansen, Dieke Postma, Rasmus Jakobsen

**Affiliations:** † Department of Civil, Structural and Environmental Engineering, Trinity College Dublin, College Green, Museum Building, D02 PN40 Dublin 2, Ireland; ‡ Department of Plant and Environmental Sciences, University of Copenhagen, DK-1871 Copenhagen, Denmark; § Bayerisches Geoinstitut, University of Bayreuth, 95440 Bayreuth, Germany; ∥ School of Earth Sciences, University of Bristol, BS8 1RJ Bristol, U.K.; ⊥ Department of Chemical and Biochemical Engineering Bio Conversions, Technical University of Denmark, DK-2800 Lyngby, Denmark; # Nordic Center for Earth Evolution, Institute of Biology, University of Southern Denmark, DK 5230 Odense M, Denmark; ¶ GEUS, Geological Survey of Denmark and Greenland, DK-1350 Copenhagen, Denmark

**Keywords:** isotope exchange, Fe-(oxyhydr)oxides, radioisotopes, kinetic assemblage, Fe^2+^_aq_, contaminant mobilization, aquatic ecosystems

## Abstract

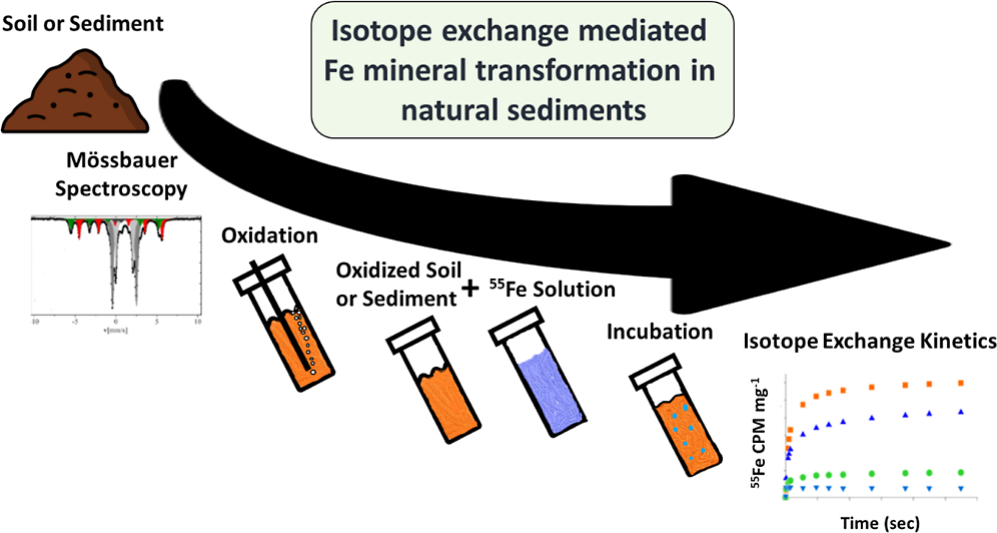

The catalytic effect of aqueous Fe(II) (Fe^2+^_aq_) on the transformation of Fe(oxyhydr)oxides has been extensively studied in the laboratory. It involves the transfer of electrons between Fe^2+^_aq_ and Fe-(oxyhydr)oxides, rapid atomic exchange of Fe between the two states, and recrystallization of the Fe-oxides into more stable Fe-(oxyhydr)oxides. The potential occurrence of these reactions in natural soils and sediments can have an important impact on biogeochemical cycling of iron, carbon, and phosphorus. We investigated the possible isotopic exchange between Fe^2+^_aq_ and sedimentary Fe(III) in Fe–Si–C-rich lake sediments. ^57^Fe Mössbauer spectroscopy was used to evaluate Fe mineral speciation in unaltered lake sediments. Unaltered and oxidized sediment laboratory incubations were coupled with a classical kinetic approach that allows a quantitative description of the reactivity of assemblages of Fe-(oxyhydr)oxides found in sediments. Specifically, unaltered and oxidized sediment samples were separately incubated with an ^55^Fe^2+^_aq_-enriched solution and exchange was observed between ^55^Fe^2+^_aq_ and sedimentary Fe(III), highest in the top of the sediment and decreasing with depth with the ^55^Fe^2+^_aq_ tracer distributed within the bulk of the sedimentary Fe(III) phase. Our results indicate that atomic exchange between Fe^2+^_aq_ and sedimentary Fe(III) occurs in natural sediments with electrons transferred from the Fe(III)-particle to Fe(III)-particle via Fe^2+^_aq_ intermediates.

## Abstract 2

This study illustrates atomic exchange between Fe^2+^_aq_ and sedimentary Fe(III) in natural sediments potentially controlling the mobility of P and other contaminants binding to Fe-(oxyhydr)oxides.

## Introduction

Iron oxyhydroxide mineral phases, hereafter called Fe-(oxyhydr)oxides, show a highly dynamic behavior as illustrated by the occurrence of different Fe-(oxyhydr)oxide minerals in natural environments.^1^ In anoxic soils and sediments, Fe-(oxyhydr)oxides and Fe^2+^_aq_ may coexist.^2^ Early laboratory studies found that this coexistence of ferric Fe-(oxyhydr)oxides and Fe^2+^_aq_ may result in the recrystallization and transformation of thermodynamically unstable short-range ordered Fe-(oxyhydr)oxides such as ferrihydrite into more stable crystalline Fe-oxides such as goethite, lepidocrocite, hematite, and magnetite.^3−6^ Furthermore, dissolved S(–II) can react with Fe(III)-containing minerals or Fe(III) minerals can undergo microbially induced reductive dissolution resulting in mineral transformation.^7−9^ Such processes can impact the reactivity and specific surface area of Fe-(oxyhydr)oxides minerals, which influence associated biogeochemical processes involved in carbon (C), phosphorus (P), nitrogen (N), and sulfur (S) cycling,^10,11^ Fe-(oxyhydr)oxide mineral-mediated cycling of trace elements,^12,13^ and degradation of organic matter (OM) in soils and sediments.^14,15^ In sediments, microbial respiration of Fe(III) (oxyhydr)oxides leads to the release of Fe^2+^_aq_. Whether the interaction of this Fe^2+^_aq_ with remaining Fe-(oxyhydr)oxide causes transformation into more stable Fe-oxides could depend on the sediment composition, e.g., the OM content.^16^

More recent laboratory studies have further demonstrated the catalytic effect of Fe^2+^_aq_ causing accelerated transformation from less stable Fe-(oxyhydr)oxides such as ferrihydrite to more stable Fe-(oxyhydr)oxides.^17−19^ If this process also occurs in natural sediments, it implies that processes taking place at the oxic/anoxic interface are much more complex than previously perceived because the first Fe^2+^_aq_ produced will cause extensive recrystallization of the remaining pool of Fe-(oxyhydr)oxide in the sediments. Considerable laboratory work under abiotic and biotic conditions has been undertaken to further understand the mechanism, processes, and parameters associated with Fe mineral phase transitions. Such studies have illustrated that the presence of anions,^20−22^ Fe^2+^_aq_/Fe(III) ratio,^19,23^ S(–II)/Fe(III) ratio,^24^ pH,^25^ and additional mineral phases^26^ are important factors influencing Fe mineral phase formation and transformation kinetics. The vast majority of these studies were carried out using synthetic Fe-(oxyhydr)oxide phases, hence it is difficult to fully appreciate the actual effects of natural complex conditions including but not limited to the impact of natural mineral assemblages, variations in natural Fe-(oxyhydr)oxide stability, and sediment microbiology.^27^ Recent work using Mössbauer spectroscopy has investigated the transformation of various synthetic iron minerals (enriched in ^57^Fe) mixed into natural soils/sediments to investigate the effects of the complex soil matrix under more natural conditions.^28^ Nonetheless, studies using natural sediments remain uncommon despite their significance for a deeper understanding of Fe mineral transformation in natural systems and associated cation and anion mobilization and cycling.

Previous studies have examined the impact of redox-induced transformations on native Fe-(oxyhydr)oxides mineral phases by exposing native soils to recurring redox oscillations.^29,30^ In addition, particular soils were collected in the field and the dynamic impact of redox conditions on their native Fe-(oxyhydr)oxides phase transitions were assessed and compared using Mössbauer spectroscopy.^31,32^ The results from these studies indicate that native Fe-(oxyhydr)oxide minerals exposed to recurring redox fluctuations can become more crystalline or less crystalline. Specifically, Fe-(oxyhydr)oxides along a redox gradient in basaltic soils were shown to decrease in crystallinity with increasing rainfall and leaching (flushing) of Fe^2+^_aq_.^31^ In contrast, soil suspension incubations illustrated less crystalline amorphous Fe-(oxyhydr)oxides transformed to more crystalline forms (e.g., goethite or hematite) with no Fe^2+^_aq_ leaching.^30^ A previous study illustrated the role vivianite plays in P retention within this in natural Si–Fe–C-rich lake sediment,^33^ and here we show its concurrent release with Fe reactivity experiments indicating P association with reactive Fe-(oxyhydr)oxide minerals.^34^

We demonstrate that isotopic exchange between Fe^2+^_aq_ and Fe(III)-phases occurs not only with pure Fe-(oxyhydr)oxides in synthetic systems but also with Fe(III)-phases in natural Si–Fe–C-rich lake sediment using ex-situ microcosm incubation experiments. As a consequence of the Fe^2+^_aq_/Fe(III)-phase atomic exchange, electrons shuttle between the aqueous and solid phases and thereby provide a mechanism for electron transport through sediments. We initially identified the primary Fe pool in the upper lake sediment profile using Mössbauer spectroscopy and applied a kinetic approach to categorize the likely Fe-(oxyhydr)oxide phases present. We incubated both unaltered and oxidized Fe–Si–C-rich lake sediment with ^55^Fe^2+^_aq_. Subsequently, a reductive kinetic approach coupled with measurement of released ^55^Fe^2+^_aq_ activity was used for a quantitative description of the reactivity of the assemblages of Fe-(oxyhydr)oxide found in the sediments which were involved in the Fe^2+^_aq_/Fe(III) exchange process. This experimental approach could be applied relatively easily to other natural sediment environments, opening alternative avenues for studying Fe mineral transformations in soils and sediments and potentially the impact on phosphate and heavy metal dynamics.

## Materials and Methods

### Study Area and Sediment Sampling

The sediments were collected in Lake Ørn, located southwest of the urban area of Silkeborg, central Jutland, Denmark. Lake Ørn is a shallow eutrophic freshwater lake situated in a glacial landscape. The lake receives a high input of iron in the form of Fe(III) precipitates from the primary tributary Funder Å, estimated at 45 g Fe m^–2^ yr^–1^^35^^,^.^36^ Sediment cores (50 and 7.4 cm diameter) were collected in February 2010 using a gravity corer (Kajak sampler). Cores were processed within a few hours in the laboratory near the in situ temperature of 3–4 °C in a cold room in an effort to preserve the natural mineral assemblage or groupings within the lake sediment profile. To obtain porewater and sediment samples, the sediment cores were inserted into a N_2_-filled portable glovebag through an airlock at the bottom of the bag and sliced into 1 cm sections at appropriate intervals. Sediment was placed in 15 or 50 mL polypropylene centrifuge tubes which were sealed tightly. The composition of the porewater was determined using methods described previously.^36,37^ The experimental design is summarized in Figure S6.

### Characterization of Unoxidized Sediments

Unoxidized sediment samples were analyzed in a previous study using X-ray diffraction with Co–Kα radiation, scanning electron microscopy, and transmission electron microscopy (TEM).^33^ In the present study, samples from depths of 1–2 and 9–10 cm below the lake bottom were additionally analyzed using Mössbauer spectroscopy at 80 and 4 K using a Janis continuous flow cryostat operated with liquid helium. Spectra were collected in transmission mode on a constant acceleration Mössbauer spectrometer equipped with a nominal 1.85 GBq ^57^Co source in a Rh matrix. Velocity scales were calibrated using Fe foil at room temperature, and spectra were fit using Recoil software (University of Ottawa, Ottawa, ON, Canada).

### Sediment Leaching, Incubations, and Radioisotope Methodology

All handling of the samples was done inside an anoxic glovebox (Coy Laboratory Products), purged with a mixture of 95% N_2_ and 5% H_2_. All experiments were biotic and were carried out using natural lake sediment. Leaching experiments of sediments (stored frozen at −80 °C within N_2_-filled bags) with HCl and ascorbic acid were carried out in a 250 mL reaction vessel equipped with an automatic titrator to maintain a constant pH of 3 in the suspension. For each sediment sample, two parallel leaching experiments were carried out (0.2 g sediment/100 mL solution); one with 1 mM HCl solution (pH 3) and one with 10 mM ascorbic acid solution adjusted to pH 3 with HCl, similar to the procedures developed previously.^38^ HCl at pH 3 only releases the Fe^2+^_aq_ that can be released at pH 3 due to desorption and dissolution of, e.g., siderite and vivianite. Ascorbic acid at 10 mM and pH 3 in addition reductively dissolves Fe-(oxyhydr)oxides including ferrihydrite, lepidocrocite, and poorly crystalline goethite.^18,38,39^ The difference between ascorbic acid and HCl extractable Fe can therefore be referred to as reductively dissolved Fe(III) phases in the sediment.

For incubations of unaltered sediment, 1 mL of a 0.2 mM Fe^2+^_aq_ solution with an activity of 1.3 MBq 1 mL^–1^ (26 × 106 CPM ml-1) ^55^Fe^2+^_aq_ was added to ∼90 mL of fresh sediment which represents approximately 2900 CPM mg^–1^. The added Fe^2+^_aq_ is insignificant compared to the Fe^2+^_aq_ present in the porewater, which varies from 0.1 to 0.6 mmol L^–1^. This appears to give a low Fe^2+^_aq_/Fe(III) ratio compared to studies on synthetic systems; however, in our natural sediments loosely bound/sorbed Fe^2+^_aq_ is presumably also part of the Fe^2+^_aq_/Fe(III) exchange. For the 1–2 cm sediment section, the Fe(III) content of 1440 mmol kg^–1^ can be compared with the Fe^2+^_aq_ released (after 30 s) which was 310 mmol kg^–1^. This translates to 322 mmol L^–1^ Fe^2+^_aq_ and the Fe^2+^_aq_/Fe(III) ratio is 0.22 which is similar to the ratios used in the studies by Pedersen et al. 2005. For the oxidized sediment experiment (described below). the activity of the tracer was ten times higher to take the larger pool of Fe-(oxyhydr)oxides into account. After adding the ^55^Fe^2+^_aq_ solution, mixing was carried out with a magnetic stirring bar for 2 h in case the addition of the tracer had led to local Fe-mineral precipitation. In a 100 mL syringe, about 90 mL of isotope-laden sediment was taken up for further incubation. A small stirring bar was placed into the syringe along with the sediment when the incubations were started and when mixing was required it was held over a stirring plate. Approximately 8 mL of sediment was expelled from the syringe at each sampling time; the exact amount was determined by weighting. The sediment was washed free of the added ^55^Fe^2+^_aq_ solution, and part of the porewater and sediment Fe^2+^_aq_, by immersion in 50 mL of a deoxygenated 1 mM HCl, 500 mM MgCl_2_ solution and shaking for 10 min, followed by centrifugation. This step was carried out three times, and the sediment was thereafter frozen at −80 °C for later processing. After thawing a sediment sample, parallel leaching experiments were carried out with 10 mM ascorbic acid and 1 mM HCl at a constant pH of 3, adding approximately 0.2 g of sediment/100 mL solution, while monitoring ^55^Fe^2+^_aq_ and bulk Fe release. ^55^Fe^2+^_aq_ activity was measured by adding 500 μL of sample to 4.5 mL of scintillation cocktail. The ^55^Fe^2+^_aq_ activity was determined by liquid scintillation counting on a Wallac Win Spectral 1420 liquid scintillation counter. The counting efficiency was 0.36. The difference in ^55^Fe^2+^_aq_ activity between the ascorbic acid and HCl leachates was taken as the ^55^Fe^2+^_aq_ activity released by reductive dissolution from Fe(III) phases in the sediment.

For one experiment, surface lake sediment was artificially oxidized to remove possible artifacts of naturally present Fe(II). The 0–2 cm sediment sections from the three cores were pooled and homogenized. The sediment was then divided over two beakers and stirred vigorously for 48 h while bubbling with air at a rate of 30 mL/min. Thereafter, the sediment was deoxygenized by bubbling with N_2_, while stirring vigorously, for 48 h. Subsequently, the sediment was incubated and sampled, as described above.

## Results

Sediment geochemistry in Lake Ørn. The lake sediments at the sampling site are organic rich and become anoxic within 1–2 mm below the sediment surface.^33^Figure S1 summarizes the porewater chemistry in the uppermost sediments. The degradation of OM coupled with suppressed nitrification due to a lack of O_2_ is indicated by the increase of NH_4_^+^ over depth, reflecting the release of N-components from sedimentary OM. The steep increases in Fe^2+^_aq_ and alkalinity with sediment depth indicate Fe(III) reduction is taking place, though this was not measured directly. Because of the abundance and deep penetration of highly reactive Fe(III)-phases in winter and spring, sulfate reduction is suppressed over most of the 0–10 cm depth range that is studied here.^36^

**Figure 1:**
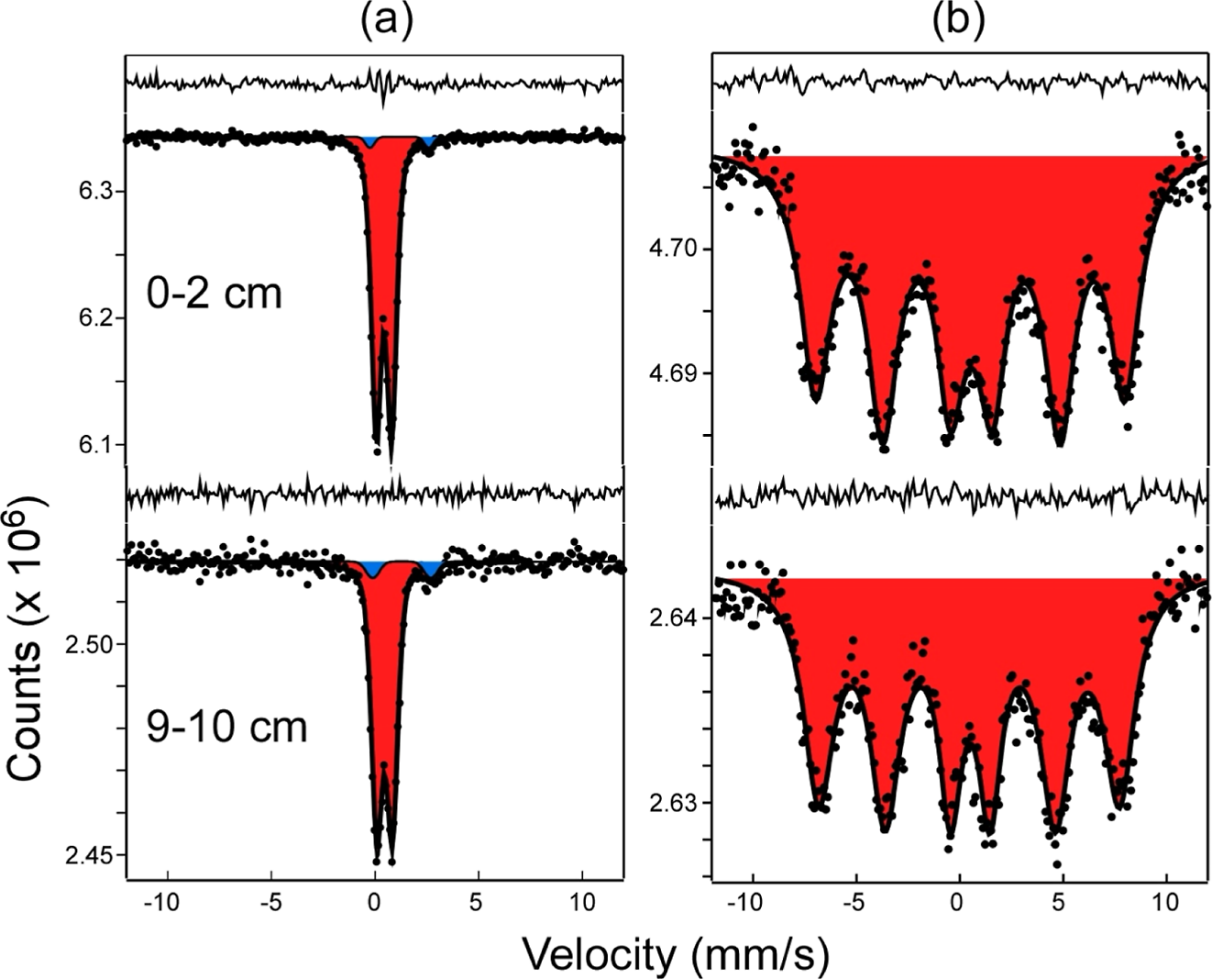
Mössbauer spectra of sediments from the bottom of Lake Ørn recorded at (a) 80 K and (b) 4 K. Spectra at 80 K were fit to doublets assigned to Fe^3+^ (red) and Fe^2+^ (blue). Spectra at 4 K were fit to a single Fe^3+^ component with a rapid fluctuating magnetic field. Fe^2+^ absorption was too small to be resolved in 4 K spectra. The fit residual is shown above each spectrum.

Previous mineralogical analysis of the sediments identified vivianite (by XRD) and possibly a poorly crystalline clay silicate that was tentatively classified as the phyllosilicate hisingerite (by TEM).^33^ Mössbauer spectroscopy at room temperature and 80 K showed the sediment Fe(III)/Fe(II) ratio to decrease with increasing depth but was unable to provide definite identification of any Fe(III) phases.^33^ The distribution of Fe in the sediment, using various chemical extraction methods (Figure S1), showed a downward decrease in oxalate-extractable-Fe and an increase in the Fe^2+^_aq_ content. Our new Mössbauer data support the observed increase in Fe^2+^_aq_ with depth and additionally provide insight into Fe(III) speciation through measurements at 4 K. Spectra can be fit to a single Fe^3+^ component with rapidly fluctuating magnetic field (Figure 1) that is diagnostic of small particle size (<10 nm) and association with nonmagnetic elements such as C and Si. The reduced hyperfine magnetic fields (46 ± 1 T) and low quadrupole shifts (−0.03 ± 0.03 mm/s) are strongly characteristic of a ferrihydrite-like phase.^40^ In addition, the 4 K Mössbauer spectrum of an amorphous Fe-(oxyhydr)oxide phase identified as Fe(OH)3.0.9H_2_O from freshwater springs has nearly identical hyperfine parameters as the spectra from our study.^40^ Hence, it is likely that the dominant Fe-oxide phase within the sediment profile is an X-ray amorphous small-particle Fe-(oxyhydr)oxide that is bound to OM or associated with Si.

**Figure 2:**
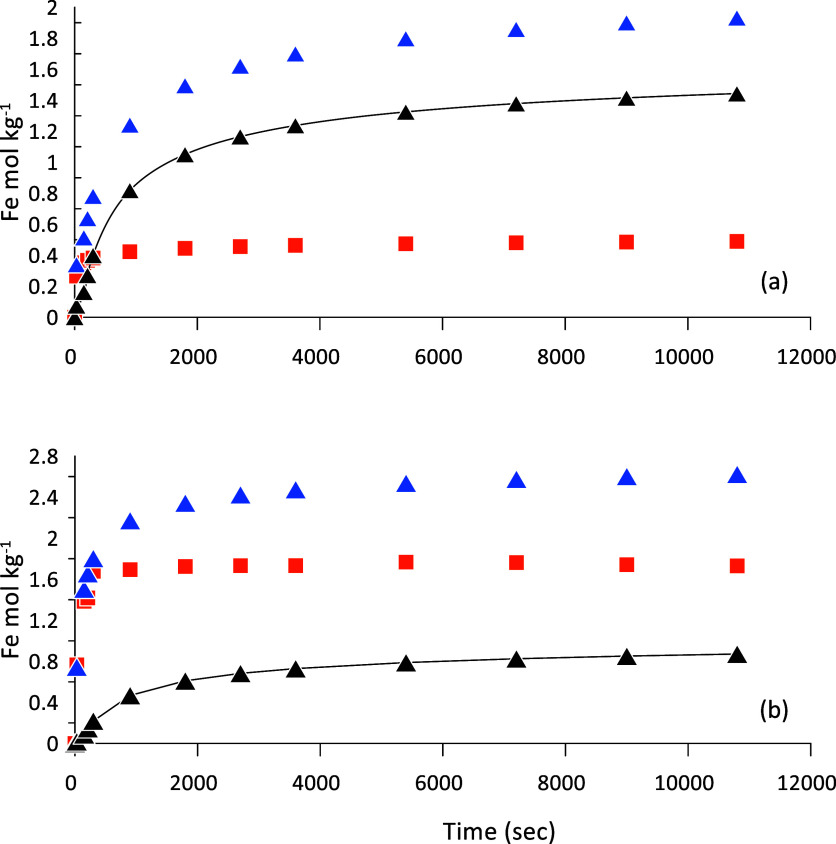
Release of iron from Lake Ørn unoxidized sediment by parallel leaching with 1 mM HCl (■-orange) and 10 mM ascorbic acid (▲-blue), both at a constant pH of 3. The difference, given by the bold line (−) and (▲), is attributed to reductive dissolution of Fe(III) phases. Panel (a) is for 1–2 cm and (b) for 9–10 cm depth.

The speciation of iron in the sediment was also assessed by parallel leaching experiments with HCl and 10 mM ascorbic acid, both at pH 3. The difference between the ascorbic acid and HCl releases quantifies easily reducible Fe-(oxyhydr)oxide.^34,38^ There was a very fast initial release of Fe^2+^_aq_ by HCl and thereafter the Fe concentration quickly reached a constant level (Figure 2). In the uppermost sample (1–2 cm depth, Figure 2a) the Fe(II) concentration reaches 0.5 mol/kg, while in the lower sample (Figure 2b) Fe(II) reaches 1.7 mol/kg. Ascorbic acid leaches up to 2.0 mol/kg of Fe in the uppermost sample and about 2.6 mol/kg in the lower sample (Figure 2). These sediments are indeed extremely rich in iron. For comparison, the total Fe content of the sediments is close to 3 mol/kg and quite constant (Figure S1), and most Fe is accordingly present in a highly reactive form. The release of easily reducible sedimentary Fe(III) is shown by the thick line in Figure 2. The reactivity of Fe(III) in the sediment was estimated by fitting the data for reducible Fe(III) to the rate expression.
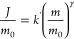
1Here, *J* is the overall reduction rate (mol s^–1^), *m*_0_ is the initial sum of reactive Fe(III) minerals, and *m* is the remaining mass at a given time (both in mol). The reactivity is given by the parameters *k*′, which is the initial rate (s^–1^), and γ. In natural sediments, γ characterizes the reactivity distribution in a reactive continuum of dissolving Fe(III) phases.^10,34,38^ As described previously, the reactivity of Fe(III) phases in natural sediments based on the rate law (eq 1) can be obtained by fitting of the three parameters; *m*_0_, *k*′, and γ.^38^ The obtained values are *k*′ = 11 × 10–4 s^–1^, γ = 1.82 at 1–2 cm and *k*′ = 9 × 10–4 s^–1^, γ = 1.89 at 9–10 cm depth. The initial rates are slightly higher than those reported for synthetic ferrihydrites (*k*′ = 3–8 × 10–4 s^–1^, γ = 0.75–2.3),^18,38,39^ supporting the conclusion from Mössbauer spectroscopy indicating the presence of X-ray amorphous Fe-(oxyhydr)oxides.

### Isotope Exchange between Aqueous ^55^Fe^2+^_aq_ and Sedimentary Fe(III)

**Figure 3:**
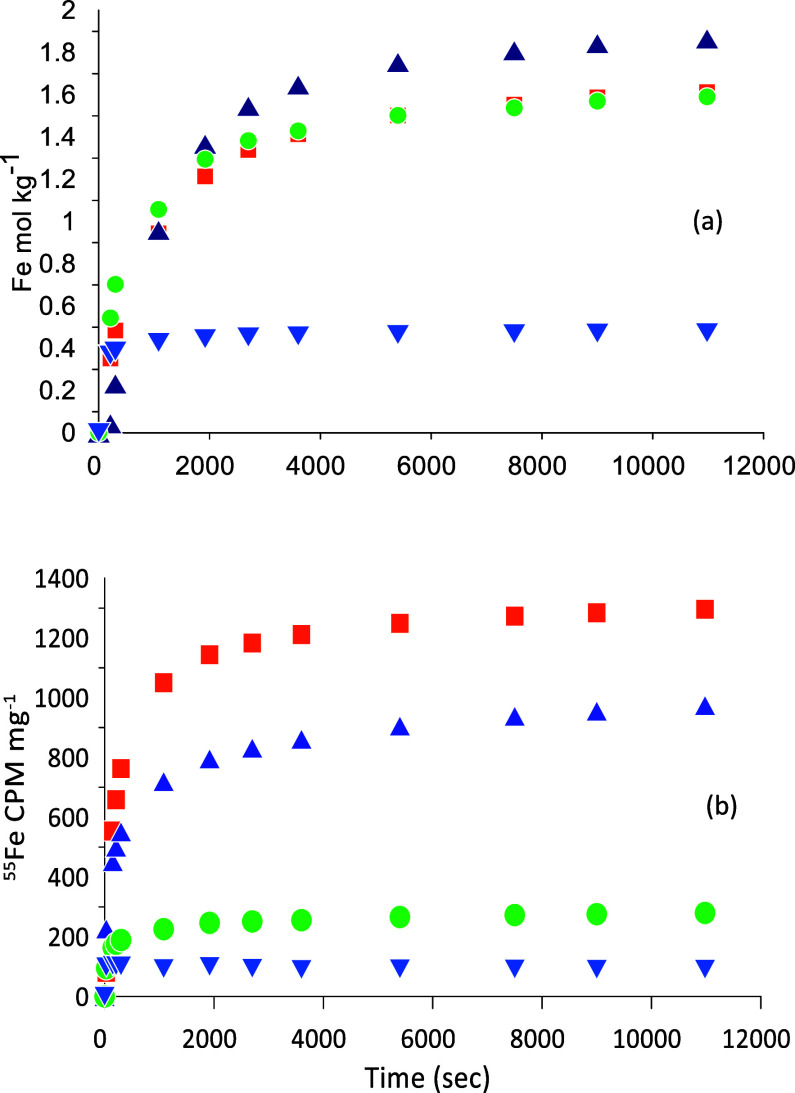
Release of (a) Fe^2+^_aq_ and (b) ^55^Fe^2+^_aq_ CPM mg^–1^ by reductive dissolution with ascorbic acid, subtracted by the release by HCl, from Lake Ørn sediments after 21 h of incubation with a ^55^Fe^2+^_aq_-labeled solution. The symbols reflect the following depth ranges: 0–2 cm ■ (orange), 2–4 cm ▲ (blue), 4–6 cm ● (green), and 8–10 cm▼ (blue).

To examine our hypothesis that electron transfer between aqueous Fe^2+^_aq_ and solid-phase Fe(III) also occurs in natural sediments, we designed experiments where an ^55^Fe^2+^_aq_-enriched solution was incubated together with lake sediment from different depths. Subsequently, we investigated to what extent the ^55^Fe^2+^_aq_ tracer had become incorporated in the sedimentary Fe(III) phase and afterward could be released by reductive dissolution with ascorbic acid. The procedure is summarized in Figure S6. The results in Figure 3 are given for 21 h of incubation. Additional results for 45 h of incubation (Figure S2) show the same general trends.

Figure 3a shows the release of Fe(III) from the sediment by reductive dissolution with ascorbic acid. The most reactive Fe(III) is present in the top 6 cm of the sediment, while the 8–10 cm sample has a much less reactive Fe(III). Overall, the results are comparable to those presented in Figure 2. As Fe(III) is released from the sediment by reductive dissolution with ascorbic acid, ^55^Fe^2+^_aq_ is also freed (Figure 3b). Because the sediment had been thoroughly washed before reductive dissolution and any remaining Fe^2+^_aq_ released by HCl-leaching is subtracted, this must imply that part of the ^55^Fe^2+^_aq_ originally present in the aqueous phase has become incorporated in a sedimentary Fe(III) phase and is not released until the Fe(III) phase is reductively dissolved. The concomitant release of ^55^Fe^2+^_aq_ and bulk Fe during reductive dissolution was also observed by Pedersen et al. (2005) for synthetic Fe-(oxyhydr)oxides and suggests that isotopic exchange between aqueous ^55^Fe^2+^_aq_ and solid-phase Fe(III) has occurred.^39^

**Figure 4:**
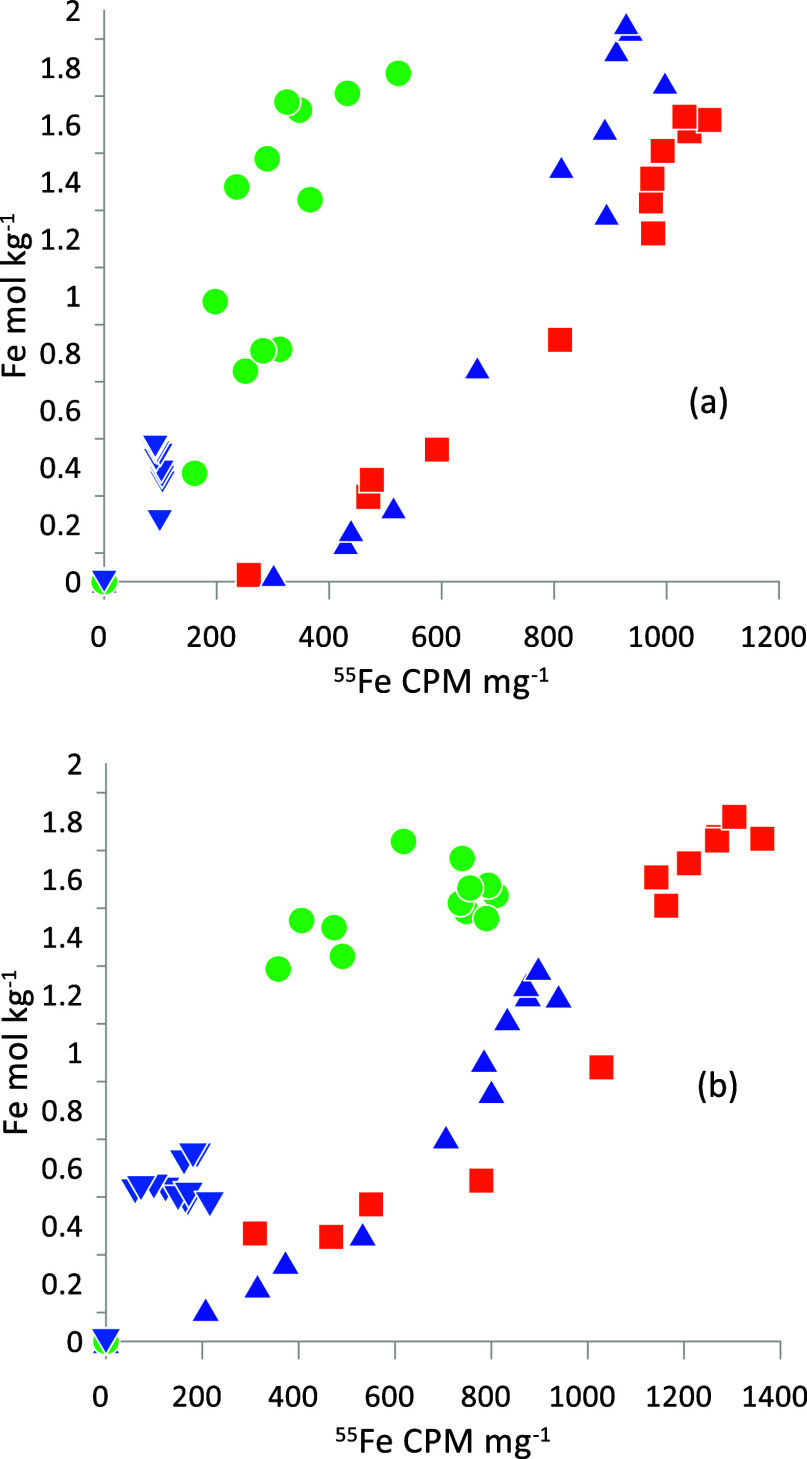
Stoichiometry of Fe^2+^_aq_ and ^55^Fe^2+^_aq_ CPM mg^–1^ release by reductive dissolution, subtracted by the release by HCl, from Lake Ørn sediments after (a) 21 and (b) 45 h of incubation with a ^55^Fe^2+^_aq_ solution. The time dependency for 21 h is shown in Figure 3 and for 45 h is displayed in Figure S2. The symbols reflect the following depth ranges: 0–2 cm ■ (orange), 2–4 cm ▲ (blue), 4–6 cm ● (green), and 8–10 cm▼ (blue).

Comparison of the curves for Fe and ^55^Fe^2+^_aq_ in Figure 3 shows differences in their relative releases. Therefore, Figure 4 compares the stoichiometry of the releases of Fe and ^55^Fe^2+^_aq_. In the 21 h incubation data (Figure 4a), there is a marked difference between the 0–2 and 2–4 cm depth samples compared to the 4–6 and 8–10 cm depth samples. The 0–2 and 2–4 cm samples show ^55^Fe^2+^_aq_ release of about 81 and 501 CPM mg^–1^, respectively, coming out with the first of the small amounts of bulk Fe, and this amount of tracer is presumably associated with the surface. Thereafter, a near constant stoichiometry of Fe^2+^_aq_ and ^55^Fe^2+^_aq_ release is found, indicating that the tracer is incorporated into the bulk of the Fe(III) phase and is released gradually when this phase is being reductively dissolved by ascorbic acid. In the deeper, 8–10 cm depth samples, little ^55^Fe^2+^_aq_ has been taken up by the solid, and most of what is there comes out with the release of the first iron and is apparently surface associated. A minor part of the difference between the uptake in the upper and lower samples could be related to a lower ^55^Fe^2+^/Fe^2+^_aq_ ratio in the pore water. In the 45 h incubation (Figure 4b), particularly the deeper sediment has taken up more ^55^Fe^2+^_aq_ tracer, so the difference between the shallower and the deeper samples has become smaller. Apparently, the samples with less reactive Fe require more time to incorporate the ^55^Fe^2+^_aq_ tracer.

### Isotope Exchange with Artificially Oxidized Surface Sediment

Because there is so much total Fe(II) in the sediment (the immediate Fe release from the nonreducing HCl leaching seen in Figure 2) as dissolved Fe^2+^_aq_, exchanged Fe(II)X_2_ and possibly siderite and/or vivianite, and because exchange between these pools of Fe^2+^_aq_ and Fe(II)_solid_ in the sediment and the added aqueous ^55^Fe^2+^_aq_ is to be expected, we were concerned whether the results in Figure 3 could be an artifact of ^55^Fe^2+^_aq_ being released from the Fe(II) pools in the sediment. Therefore, we oxidized Fe^2+^_aq_ in a sediment sample from 0 to 2 cm depth by prolonged bubbling with air. Afterward, the oxidized sediment was carefully made anoxic and then incubated with aqueous ^55^Fe^2+^_aq_. At different times, samples were taken, washed free of ^55^Fe^2+^_aq_, and subsequently leached with HCl and ascorbic acid during the release of Fe^2+^_aq_ and ^55^Fe^2+^_aq_.

**Figure 5:**
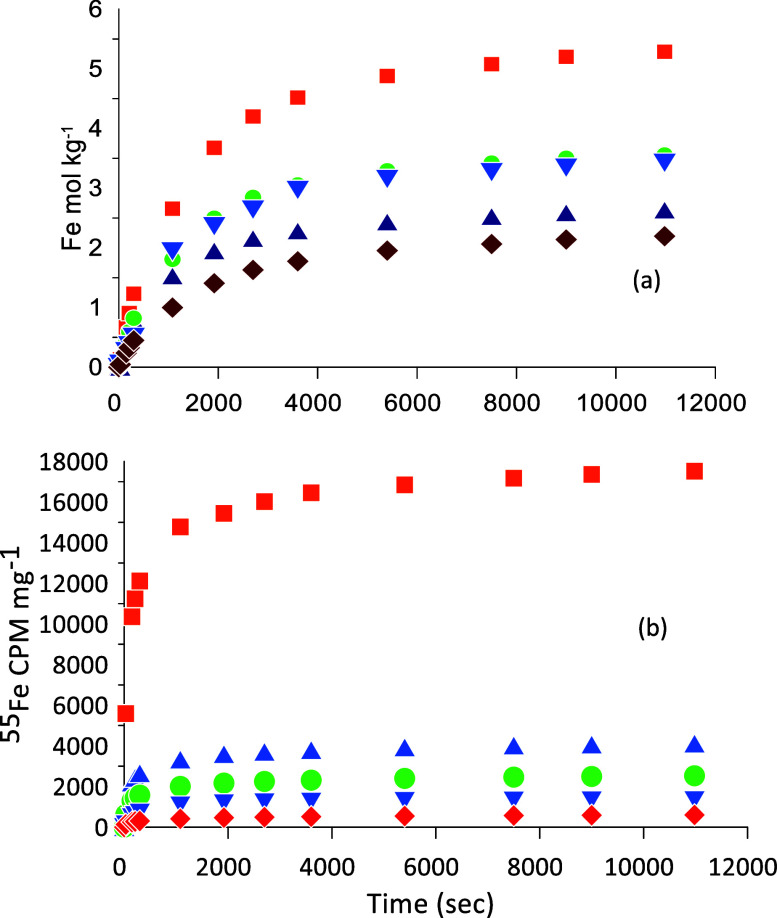
Release of (a) Fe^2+^_aq_ and (b) ^55^Fe^2+^_aq_ by reductive dissolution, subtracted by the release by HCl, from oxidized surface layer sediments (0–2 cm) from Lake Ørn after incubation with ^55^Fe^2+^_aq_. The symbols reflect incubation time: 2 h ■, 25 h ▲, 74 h ●, 290 h▼, and 458 h ⧫.

Figure 5a shows that the freshly oxidized sediment (2 h) has a very high content of highly reactive Fe(III) which is more than twice the amount measured in the nonoxidized 1–2 cm sample of Figure 2. The fitted rate constants, *k*′ = 8–9 × 10–4 s^–1^ and γ = 1.74 remain of the same order as values reported for ferrihydrite.^18,38,39^ After 74 h of anoxic incubation, the pool of Fe(III) that can be reductively dissolved by ascorbic acid has decreased by about one-third, and after 458 h, less than half is left. For unclear reasons, the results for 25 h fall outside the general trend as too much reactive Fe(III) already has been lost. The decrease in the pool of Fe(III) that can be reductively dissolved by ascorbic acid as a function of incubation time can either be due to reduction of Fe(III) and accumulation of Fe^2+^_aq_ in the sediment or recrystallization of Fe(III) into a form that is inaccessible for ascorbic acid, for example, catalyzed by the newly formed Fe^2+^_aq_. Unfortunately, we cannot quantify the amount of sedimentary Fe^2+^_aq_ formed because part of it has been lost in the ^55^Fe^2+^_aq_ washing steps. The large decrease in ascorbate-reducible Fe(III) between 2 and 25 h of incubation therefore indicates a rate of Fe(III) reduction exceeding 1 mol Fe(III) kg^–1^ d^–1^ or comprehensive recrystallization of the initially precipitated Fe(III)-phase into more stable phases which do not dissolve extensively in 10 mM ascorbic acid or a combination of the two. An Fe(III) reduction rate of 1 mol Fe(III) kg^–1^ d^–1^ corresponds to a carbon oxidation rate of 50 μmol cm^–3^ d^–1^, and this value is more than 2 orders of magnitude higher than the measured rates which were in the range 0.1–0.2 μmol cm-3 d^–1^ (to be reported elsewhere), suggesting that recrystallization is an important process. This is further indicated by the observation that after only two h 55% of the added ^55^Fe^2+^_aq_ tracer has become incorporated in the ascorbic acid-reducible fraction. As the sediment is incubated longer, less Fe and less ^55^Fe^2+^_aq_ are released and at relatively constant stoichiometric ratios. However, at all times a considerable amount of ^55^Fe^2+^_aq_ is released at a proportion higher than what was shown for the unoxidized samples in Figure 3 and also when the 10 times higher added ^55^Fe^2+^_aq_ activity is considered.

Overall, the oxidized sample shows an Fe–^55^Fe^2+^_aq_ release pattern that is very similar to what was observed for the unoxidized samples (Figure 3), which again suggests that the ^55^Fe^2+^_aq_ released is derived from the Fe(III) phase and therefore reflects the incorporation of the ^55^Fe^2+^_aq_ tracer, added as Fe^2+^_aq_, into the solid-phase Fe(III). This again indicates that isotopic exchange between aqueous ^55^Fe^2+^_aq_ and the Fe(III)-solid phase has taken place in the naturally complex sediment.

## Discussion

### Isotopic Exchange between Aqueous Fe^2+^_aq_ and Solid-Phase Fe(III)

Our results show that isotopic exchange between aqueous Fe^2+^_aq_ and an Fe(III) solid phase occurred in the exceedingly iron-rich lake Ørn sediments (Figure S1)^33^ and appears to indicate Fe^2+^_aq_-induced atomic Fe-exchange in natural lake sediments. Mössbauer spectroscopy on sediments at 4 K prior to isotope exchange incubation experiments are consistent with the dominant Fe phase within the sediment profile being X-ray amorphous Fe-(oxyhydr)oxide, possibly ferrihydrite, which is likely bound to OM and/or Si (Figure 1). During reductive dissolution, a large amount of iron is brought into solution together with the ^55^Fe^2+^_aq_ isotope tracer, with reactivity characteristics close to those of ferrihydrite^10,18^ (Figures 3 and 4). Atomic exchange between Fe^2+^_aq_ and various Fe-(oxyhydr)oxides, as the result of interfacial electron transfer, has been demonstrated for several iron oxide minerals, including ferrihydrite. Experiments were carried out with ^55^Fe^2+^_aq_-doped Fe-(oxyhydr)oxide and demonstrated complete atomic exchange within days between aqueous Fe^2+^_aq_ and Fe-(oxyhydr)oxides for both ferrihydrite and lepidocrocite and partial exchange for goethite.^18^ Subsequently, atomic exchange has also been demonstrated for goethite^41^ and extensive exchange for hematite.^42−45^ The model of “redox-driven conveyor belt” was proposed for hematite based off the evidence of Yanina and Rosso (2008).^43^ The mechanism involved was elucidated by Williams and Scherer (2004), using Mössbauer spectroscopy, who showed that the adsorbing ^57^Fe(II) ion loses an electron to form ^57^Fe(III) which is then incorporated into Fe-(oxyhydr)oxide.^46^ The electron is transported through Fe-(oxyhydr)oxide by conduction mediated by reduction of Fe(III) sites to form small polarons that enable electron hopping from one Fe atom to the next until it reaches another surface site where Fe(III) is reduced and goes into solution as Fe^2+^_aq_.^48^ Thus, Fe-(oxyhydr)oxide is formed at one end of the crystal and dissolved at another end. The result is a total recrystallization of Fe-(oxyhydr)oxide, which has been termed a redox-driven conveyor belt.^41^ It should be noted that this mechanism of through-crystal conduction is not fully supported for all of the Fe minerals. Another plausible mechanism of Fe(II)-induced Fe(III)-(oxyhydr)oxide recrystallization particularly associated with goethite involves local conduction paths and heterogeneous deposition of isotopically distinct Fe at grain boundaries and defect sites.^48^ Electron exchange between aqueous Fe^2+^_aq_ and structural Fe(II) in clay is also possible but does not lead to atomic exchange of Fe but rather to surface precipitation of Fe(III).^49^

### Fe-(oxyhydr)oxide Mineral Reactivity and Transformation Dynamics

While electron transfer and atomic exchange reactions between aqueous Fe^2+^_aq_ and Fe(oxyhydr)oxide are well established, their observation in natural lake sediments is rarely reported. One complication could be that while laboratory studies mostly employ pure synthetic Fe-(oxyhydr)oxide, sedimentary Fe-(oxyhydr)oxides are often substituted with Al, Si, and many other elements along with a close association with OM.^16,20^ It has been found that Al-substitution inhibits the Fe^2+^_aq_-catalyzed transformation of ferrihydrite.^50,51^ Likewise, atomic exchange between Fe^2+^_aq_ and goethite is inhibited by Al-substitution.^52,53^ Furthermore, Si has been shown to stabilize ferrihydrite during Fe^2+^_aq_-catalyzed mineral transformation.^20,54^ During reductive dissolution of an Fe(III) phase with reactivity characteristics close to those of ferrihydrite, a large amount of iron is brought into solution, together with the ^55^Fe^2+^_aq_ isotope tracer. There was a concomitant release of Si and P (Figures S3 and S4) but these elements were relatively small in concentration compared to Fe. In the 9–10 cm depth sample, Si release constituted about 1% of released Fe in HCl leaching and 2% in the ascorbic acid leaching, both on a molar basis. During the leaching experiments, the Si concentration remained less than 0.7 mg/L and the solutions were accordingly always subsaturated for quartz, chalcedony, and amorphous SiO_2_. The amount of phosphate released from the sediment during these experiments was much higher but still amounted to a maximum of 10% of the released Fe on a molar basis. The leaching solutions always remained strongly subsaturated for vivianite which has been deduced to be present in the sediment and is a considerable source of phosphate.^33^

In biologically active natural soils and sediments with elevated dissolved organic matter (DOM), ferrihydrite is often precipitated with natural organic matter (NOM).^15,16^ Coprecipitation of DOM with ferrihydrite can decrease the available reactive surface area and block or inhibit the adsorption of Fe^2+^_aq_.^20,55^ Previous studies have used Fe-(oxyhydr)oxides for these C/Fe ratio investigations, and some have indicated a coprecipitated C/Fe molar ratio of 1.7 to be a transformation threshold above which ferrihydrite transformation was prevented. It has been speculated that when C/Fe > 2.8, there is likely a complete blockage of adsorption sites, which prevents Fe^2+^_aq_ adsorption at the mineral surface.^55^ While our study of natural lake sediments was not able to quantify the concentration of NOM in contact with small-particle Fe-(oxyhydr)oxide, bulk organic C/oxalate extractable Fe(III) ratios were high, 4.5–18.9 median 6.8 (Supplemental Table, T1), and while the C is not necessarily associated with Fe, it shows that exchange can occur even in organic-rich systems where inhibition might be expected based on the lab studies.^56,57^ Furthermore, it has been found that carbohydrate-rich NOM stabilizes poorly crystalline Fe(III)-(oxyhydr)oxide minerals from wetlands against Fe(II)-catalyzed reductive transformation at circumneutral pH, though without impacting Fe^2+^/Fe(III) atomic exchange that can still occur.^23^ Similarly, our study showed that despite other evidence of Si, P, and C inhibiting transformations, this did not prevent the Fe isotope exchange process in Lake Ørn sediment and this could be the reason why the Fe-(oxyhydr)oxide phase remains so poorly crystalline also deeper in the sediment profile, even though we see Fe^2+^_aq_/Fe(III) atomic exchange taking place.

### Mechanism of Electron Transport in Minerals and Sediments

The classical model for electron transport in minerals or rocks is the geobattery model for sulfidic ore bodies,^58^ where a cathodic reaction occurs in the oxidized zone, consuming electrons by the reduction of O_2_, and an anodic oxidation of Fe^2+^_aq_ to Fe(III) solid phase occurs in the anoxic zone with the electrons being transported through the semiconductive sulfide ore body. A similar model has been proposed for electron transport through the sulfidic walls of a hydrothermal Black Smoker chimney.^59^ For sediments, the geobattery model was extended into a biogeobattery^60^ model in which electron transport between the cathode and anode is mediated by either a conductive bacterial network or a combination of a bacterial network and semiconductive mineral grains like Fe-(oxyhydr)oxides.^60−65^

Our results show that isotopic exchange occurs between aqueous Fe^2+^_aq_ and an Fe(III)-solid phase in the lake sediment with reactivity properties and Mössbauer spectra resembling those of ferrihydrite. If a redox gradient exists in the sediment, this may provide an inorganic pathway for transmitting electrons from a more reduced to a more oxidized environment by letting the electrons be transferred from one Fe-(oxyhydr)oxide crystal to the next via aqueous Fe^2+^_aq_ intermediates. Because of the nature of the mechanism, the rate of electron transfer must be equal to the rate of atomic exchange between Fe^2+^_aq_ and the Fe(III) solid phase. In the unaltered 0–2 cm sediment, 40% of the added ^55^Fe^2+^_aq_ tracer ended up in the ascorbic acid-reducible Fe fraction after 21 h, while in the fully oxidized sediment the incorporated ^55^Fe^2+^_aq_ fraction was 55% after only 2 h. These numbers suggest that a major part of porewater Fe^2+^_aq_ is exchanged with the sedimentary Fe(III) phase, reflecting an equivalent electron transfer. Conversely, it should be noted that in the case of some Fe-(oxyhydr)oxides such as ferrihydrite^66^ and magnetite,^63^ some small fraction of Fe(II) can be stored in the minerals. Hence the discussion around electron transport might possibly include the rate of Fe(II) detachment from the Fe-(oxyhydr)oxides. In terms of applying the kinetic data from the reductive dissolution through our technique to interpret the electron transport mechanism or behavior in our study, we can compare the ^55^Fe^2+^ release of the 25 h oxidized sediment (*k* = 0.010) with the “bulk” Fe release (*k* = 0.015). These values are quite similar and might suggest that the ^55^Fe is being released together with the “bulk” Fe–meaning that the ^55^Fe is incorporated in the Fe-(oxyhydr)oxides and not just on the surface. In addition, if we compare the gamma values (γ) for “bulk” Fe^2+^_aq_ release of the 25 h oxidized sample (γ = 3.06) with the natural 21 h sample (γ = 2.73), the value is greater in the 25 h oxidized samples, which indicates that there is a wider range of reactivity as new, very reactive Fe-(oxyhydr)oxides formed in the oxidized sample^10^ presumably explaining why more exchange has happened.

In Lake Ørn, this electron transport mechanism could help explain the upward transport of electrons resulting from sulfide and anaerobic methane oxidation.^36,67,68^ Our data shows (Figure 3) that electron transfer is faster in the surficial sediments than in the deeper sediment with less reactive Fe(III) as described before.^23^ This is equivalent to the differences in the rate of isotopic exchange found for different Fe-(oxyhydr)oxides.^39^ In addition, direct measurements of the electron hopping rate in different Fe-(oxyhydr)oxides have been carried out.^47^ A measurable numerical value of the electron exchange rate is illustrated by the increase in isotopic exchange found with increasing incubation time (Figure 5). Isotopic exchange, and thereby electron exchange, was found to increase with the concentration of Fe^2+^_aq_, and this effect was strongest at a low (<0.6 mM) Fe(II)-concentration, while at higher concentration the stimulatory effect approached saturation.^18^ This is likely important in sediments since the Fe^2+^_aq_ concentration generally increases with depth and should favor electron exchange, opposite to the effect of the Fe(III) reactivity which decreases with depth. Finally, spatial distribution of Fe-(oxyhydr)oxide in the sediment must play an important role. The distance and diffusion of Fe^2+^_aq_ between the grains could be the rate-limiting step; in our extremely Fe-rich sediment, the distances between the small-particle Fe-(oxyhydr)oxide crystals are likely to be short, which could imply a high electron transport rate. There is also the possibility that electron transport proceeds through a bacterial network in combination with semiconductive mineral grains, as was documented in an early laboratory study.^69^ Here, bacteria of the genus Shewanella were found to organize themselves in conductive bacterial networks in which semiconductive Fe-(oxyhydr)oxides are included and electrons are transferred from one Fe-(oxyhydr)oxide crystal to the next via extracellular electron transfer. Recently, further studies have shown how electron transport proceeds through a bacterial network in combination with semiconductive mineral grains^69^ in various biogeochemical cycles including sulfur^24,70^ and nitrogen.^71^ Furthermore, studies have illustrated the role of Fe(III)(mineral)–Fe(II)–Fe(III)(mineral) cycling of (semi)conductive iron oxides in the acceleration of the flow of electrons from syntrophic bacteria to methanogens in the methanogenesis process.^72^ Both the inorganic mechanism proposed here and the results of Nakamura et al. (2010)^59^ suggest the possibility of an additional pathway for electron transport through sediments which employs the semiconductive properties of iron mineral particles,^73^ which definitely deserves further study.

### Environmental Implications

The catalytic reactions between Fe^2+^_aq_ and sedimentary Fe-(oxyhydr)oxide are likely to have a major impact on the water quality of lakes, with sediments containing elevated Fe concentrations. During winter turnover, fresh Fe-(oxyhydr)oxides are formed in iron-rich lake sediment, which extensively scavenge phosphate from the lake water. It has also been found that phosphate uptake is related to the amount of reactive iron present in the sediment.^74,75^ When the sediment sufficiently reduces to bring some Fe^2+^_aq_ into solution, this may trigger extensive recrystallization. While the fate of adsorbed phosphate during recrystallization in natural Fe-(oxyhydr)oxides sediment has not yet been quantified, it seems likely that the adsorption capacity of sediment toward phosphate will decrease when more stable Fe-(oxyhydr)oxides with lower specific surface areas are formed. As it has been established that adsorbed phosphate can inhibit Fe^2+^_aq_-induced recrystallization of synthesized ferrihydrite,^54,76^ this could act as a negative feedback, limiting recrystallization and associated phosphate release. In addition, Fe-(oxyhydr)oxides are important scavengers for trace elements in natural environments and are also used for engineered entrapment of contaminants; hence, the stability of these iron oxides is of importance to predict the fate of such contaminants. Previous laboratory studies have shown that Fe^2+^_aq_-induced recrystallization of Fe-(oxyhydr)oxide can mobilize trace metals like As, Ni, Zn, Cu, Co, and Mn.^39,52,77^ Our study successfully illustrated that Fe^2+^_aq_-catalyzed recrystallization of Fe-(oxyhydr)oxide can be examined using ^55^Fe^2+^aq combined with a kinetic assemblage approach in natural environments. We observed decreased isotope exchange between ^55^Fe^2+^_aq_ in solution and Fe-(oxyhydr)oxides with depth down the sediment profile, which could be relevant to risk assessments for Fe-(oxyhydr)oxide-associated contaminants in lakes and other freshwater systems.

## Supporting Information Available

Information on the distribution of iron in the sediments and additional results from incubation experiments (PDF)
