# Supplementary material for: Isotopic Exchange between Aqueous Fe(II) and Solid Fe(III) in Lake Sediment—A Kinetic Assemblage Approach
Source: Environ Sci Technol. 2025 Mar 11;59(11):5534–44. doi: 10.1021/acs.est.4c07369 (PMC11948465; doi:10.1021/acs.est.4c07369)
Supplement: Supplementary file 1 — es4c07369_si_001.pdf [file es4c07369_si_001.pdf]

SUPPORTING INFORMATION

Isotopic exchange between aqueous Fe(II) and solid Fe(III) in lake sediment - a kinetic assemblage approach

DAVID W. O'CONNELL [*1,2], CATHERINE MCCAMMON [3], JAMES M. BYRNE [4], MARLENE MARK JENSEN [5], BO THAMDRUP [6], HANS CHRISTIAN BRUUN HANSEN [2], DIEKE POSTMA [7], RASMUS JAKOBSEN [7]

[1] Department of Civil, Structural and Environmental Engineering, Trinity College Dublin, College Green, Museum Building, Dublin 2, Ireland.

[2] Department of Plant and Environmental Sciences, University of Copenhagen, DK-1871, Copenhagen, Denmark.

[3]Bayerisches Geoinstitut, University of Bayreuth, 95440 Bayreuth, Germany.

[4] School of Earth Sciences, University of Bristol, Bristol BS8 1RJ, United Kingdom.

[5] Department of Chemical and Biochemical Engineering Bio Conversions, Technical University of Denmark, DK-2800, Lyngby, Denmark.

[6] Nordic Center for Earth Evolution, Institute of Biology, University of Southern Denmark, DK 5230, Odense M, Denmark.

[7] GEUS, Geological Survey of Denmark and Greenland, DK-1350, Copenhagen, Denmark.

*Email: david.oconnell@tcd.ie

Number of Figures: 6

Number of Tables: 1

Number of Pages: 8

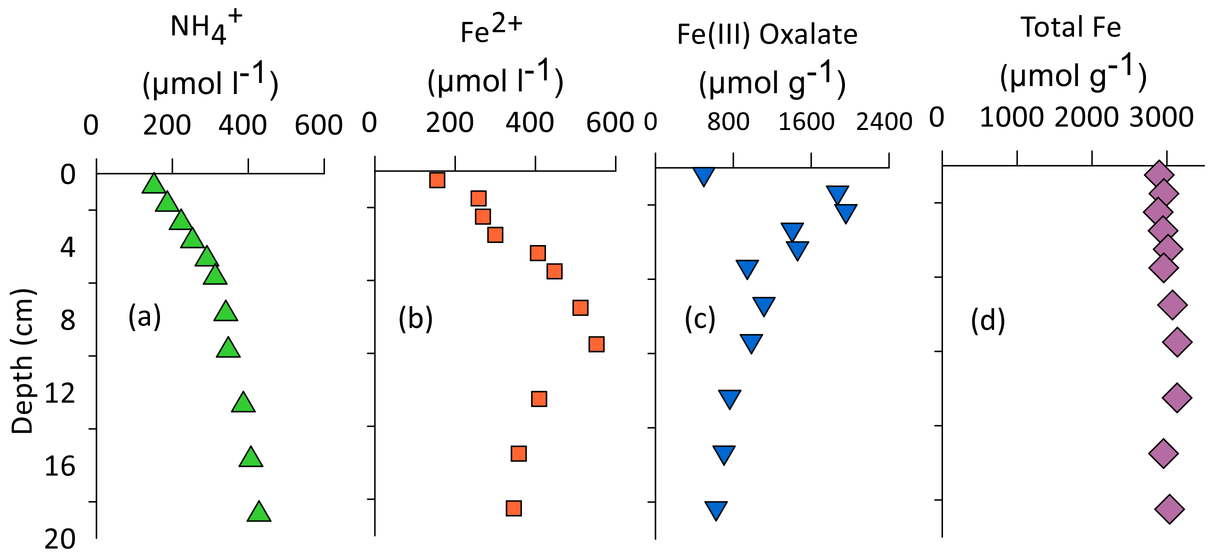

Figure S1. (a) Porewater ammonium [NH_4^+] (b) Porewater Iron [Fe^{2+}] (a), solid phase Fe(III) oxalate (b) and Total Fe (c) composition reflecting reductive dissolution of iron in Lake Ørn sediment.

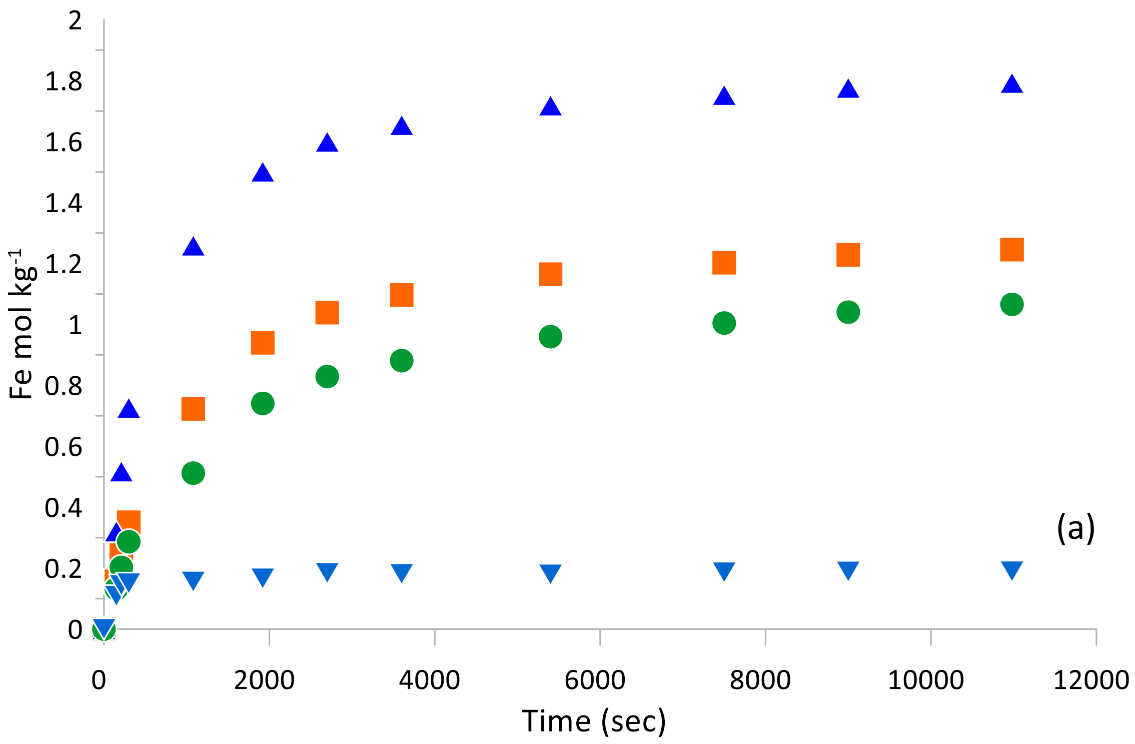
2
1.8
1.6
1.4
1.2
1
0.8
0.6
0.4
0.2
0
Fe mol kg-1
0
2000
4000
6000
8000
10000
12000
Time (sec)
(a)

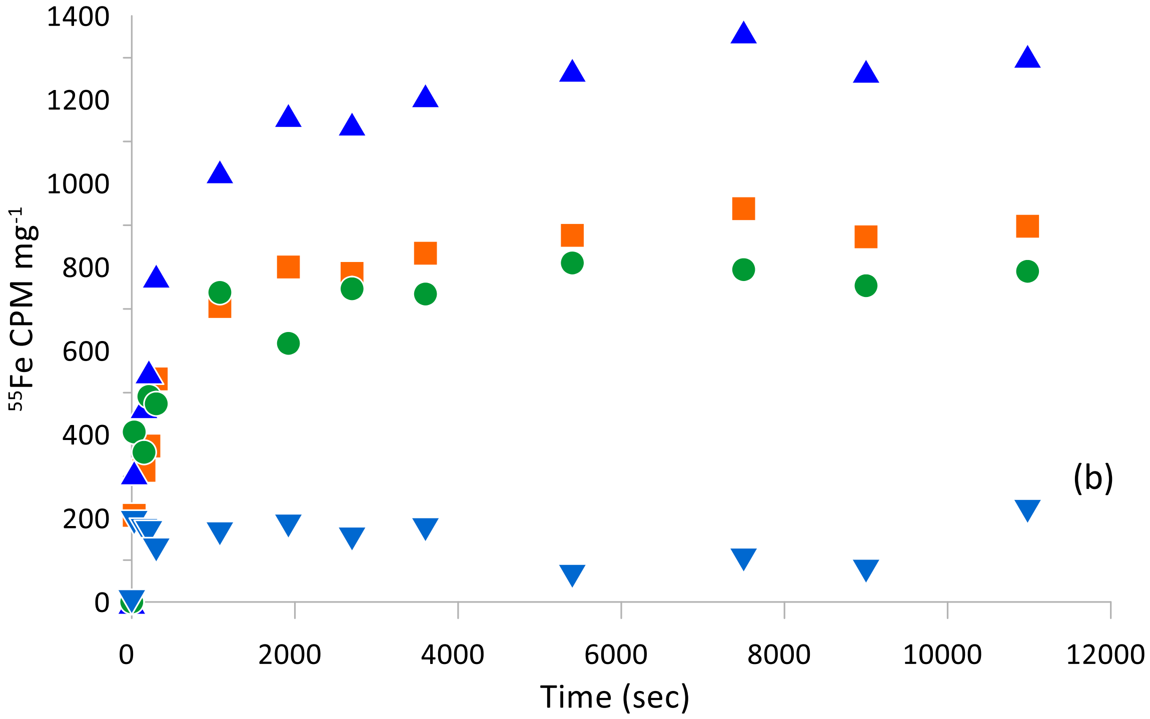

Figure S2. Release of (a) Fe [fitted] and (b) ^{55}Fe [unfitted] by reductive dissolution with ascorbic acid, subtracted by the release by HCl, from Ørnsø lake sediments after 45 hours of incubation with a $^{55}Fe(II)$ solution. The symbols reflect the following depth ranges: 0-2cm ■, 2-4cm ▲, 4-6cm ● and 8-10cm ▼.

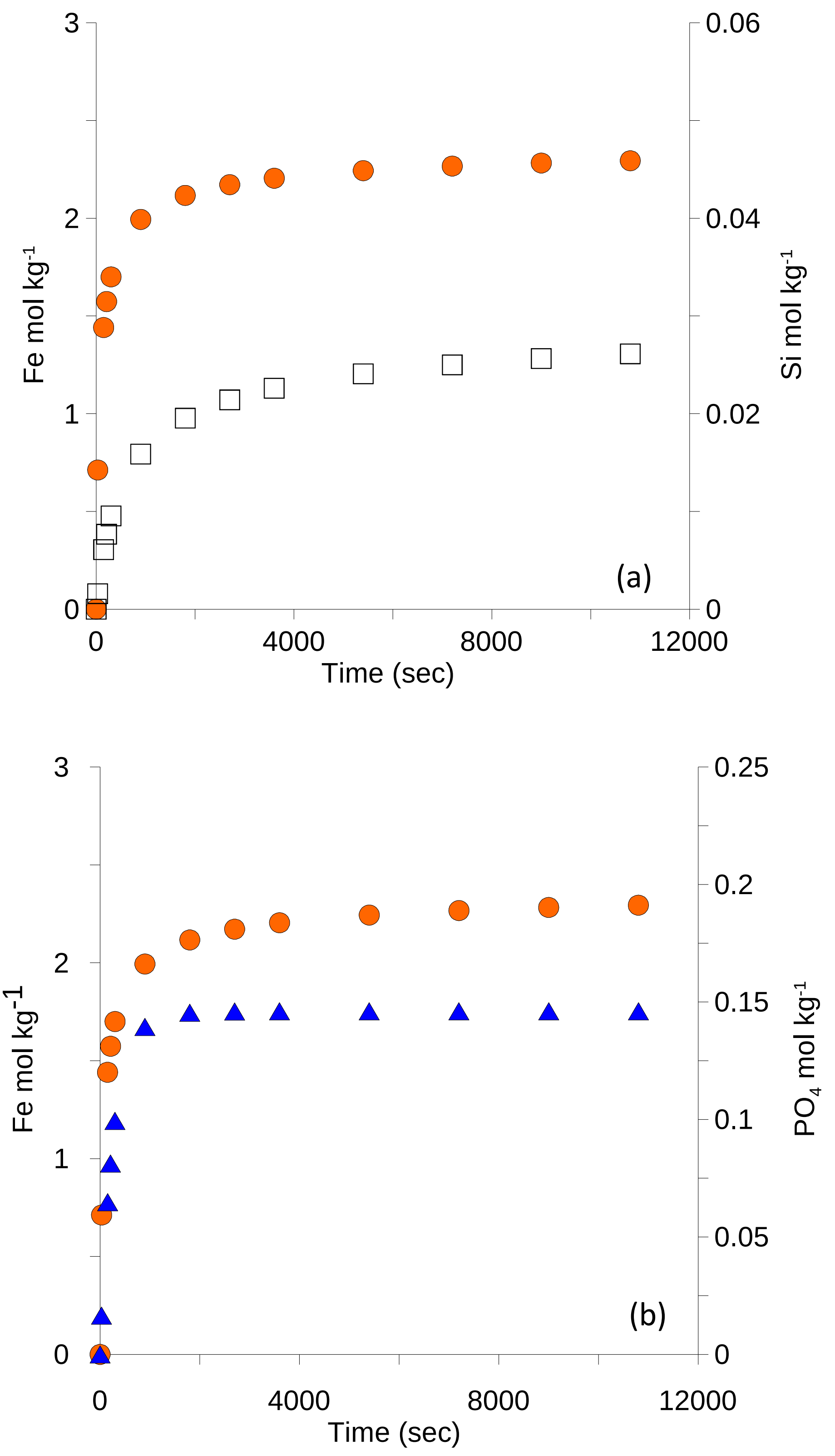

Figure S3. Simultaneous release of iron (●), silica (□) and phosphate (▲) from Ørnsø lake sediment (1-2 cm depth) by leaching with 1 mM HCl at a constant pH of 3. The data are from a different sediment core than shown in Figure 2.

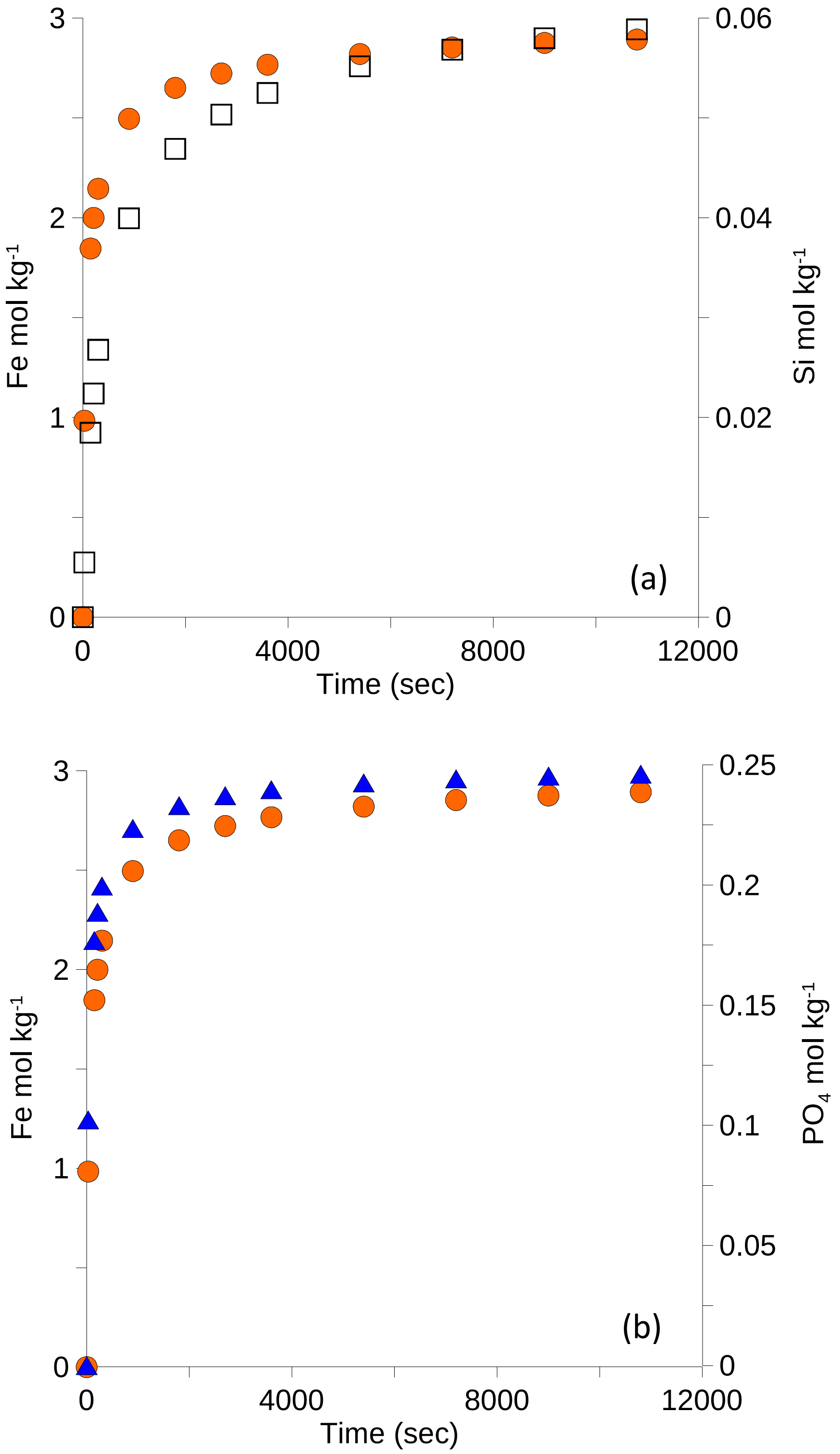

Figure S4. Simultaneous release of iron (●), silica (□) and phosphate (▲) from Ørnsø lake sediment (9-10 cm depth) by leaching with 10 mM ascorbic acid at a constant pH of 3. HCl leaching data has not been subtracted. The data are from a different sediment core than shown in Figure 2.

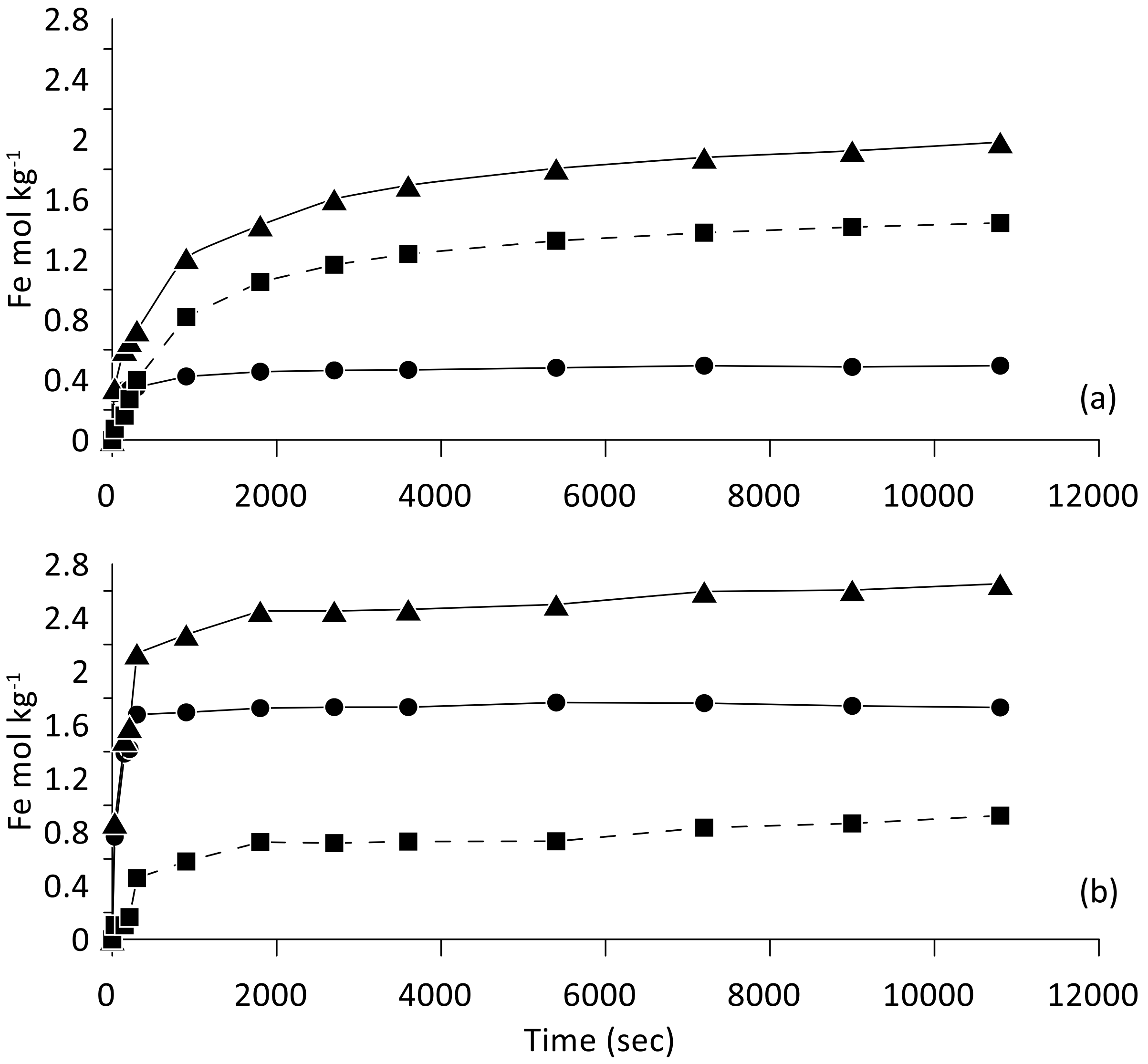

Figure S5. The (unfitted) release of iron from Lake Ørn unoxidized sediment by parallel leaching with 1 mM HCl (●) and 10 mM ascorbic acid (▲), both at a constant pH of 3. The difference, given by the broken line and square symbol (■), is attributed to reductive dissolution of Fe(III) phases. Panel (a) is for 1-2 cm and (b) for 9-10 cm depth.

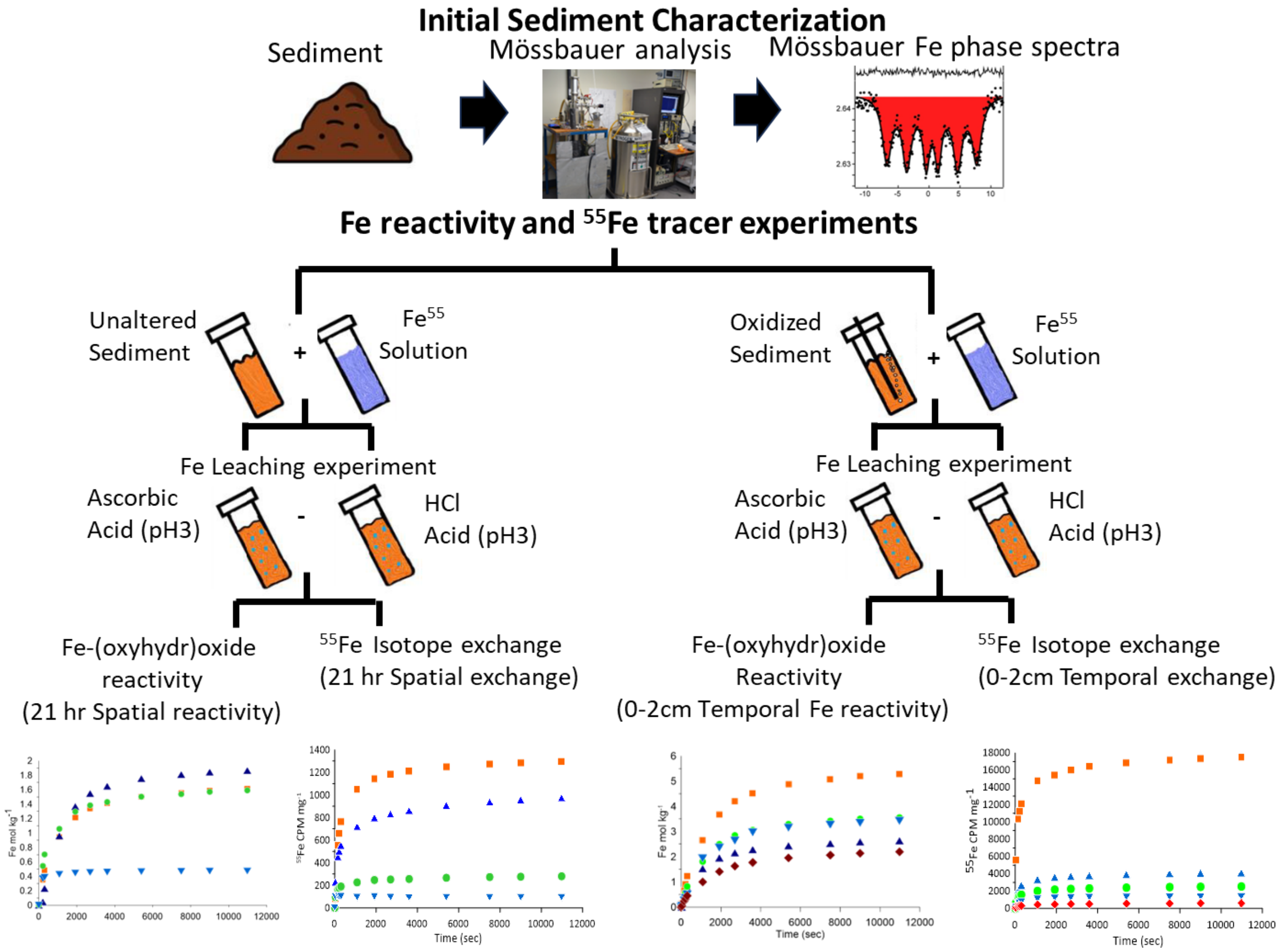

Figure S6. The conceptual figure of the experimental design for the study.

Table T1. Lake Ørn sediment Organic C [µmol g^{-1}] and Oxalate Fe(III) [µmol g^{-1}] along with the C/Fe ratio for sediment depth from 0.5 to 18.5cm.

Depth (cm)	Organic C [µmol g^{-1}]	Oxalate Fe(III) [µmol g^{-1}]	C/Fe Ratio
0.5	9398.96	496.92	18.914
1.5	8571.375	1870.73	4.582
2.5	8785.07	1956.52	4.49
3.5	7712.85	1405.96	5.486
4.5	7604.96	1459.85	5.209
5.5	8016.45	943.29	8.498
7.5	7659.76	1114.79	6.871
9.5	5977.93	985.71	6.065
12.5	5251.83	763.15	6.882
15.5	5391.43	704.84	7.649
18.5	4776.13	623.09	7.665
